# PD-L1 positivity predicts a unique hyperaggressive tumor group within MenG C meningiomas

**DOI:** 10.1093/jncics/pkag023

**Published:** 2026-03-07

**Authors:** Vijay Nitturi, Shervin Hosseingholi Nouri, Collin English, Hsiang-Chih Lu, Elizabeth Ledbetter, Diego Rojas, Sean Lau, Malcolm McDonald, Jacob J Mandel, Abdul Basit Khan, Arif O Harmanci, Akdes S Harmanci, Tiemo Klisch, Akash J Patel

**Affiliations:** Department of Neurosurgery, Baylor College of Medicine, Houston, TX, United States; Department of Neurosurgery, Baylor College of Medicine, Houston, TX, United States; Department of Neurosurgery, Baylor College of Medicine, Houston, TX, United States; Department of Pathology and Immunology, Baylor College of Medicine, Houston, TX, United States; Jan and Dan Duncan Neurological Research Institute, Texas Children’s Hospital, Houston, TX, United States; Department of Neurosurgery, Baylor College of Medicine, Houston, TX, United States; Department of Neurosurgery, Baylor College of Medicine, Houston, TX, United States; Department of Neurosurgery, Baylor College of Medicine, Houston, TX, United States; Department of Neurosurgery, Baylor College of Medicine, Houston, TX, United States; Department of Neurosurgery, Baylor College of Medicine, Houston, TX, United States; Department of Neurology, Baylor College of Medicine, Houston, TX, United States; Department of Neurosurgery, Baylor College of Medicine, Houston, TX, United States; Center for Secure Artificial Intelligence for Healthcare (SAFE), D. Bradley McWilliams School of Biomedical Informatics, University of Texas Health Science Center Houston (UTHealth), Houston, TX, United States; Department of Neurosurgery, Baylor College of Medicine, Houston, TX, United States; Jan and Dan Duncan Neurological Research Institute, Texas Children’s Hospital, Houston, TX, United States; Jan and Dan Duncan Neurological Research Institute, Texas Children’s Hospital, Houston, TX, United States; Department of Molecular and Human Genetics, Baylor College of Medicine, Houston, TX, United States; Department of Neurosurgery, Baylor College of Medicine, Houston, TX, United States; Jan and Dan Duncan Neurological Research Institute, Texas Children’s Hospital, Houston, TX, United States; Department of Otolaryngology-Head and Neck Surgery, Baylor College of Medicine, Houston, TX, United States

## Abstract

Molecular profiling has identified 3 groups of meningiomas, with MenG C tumors exhibiting the vast majority of recurrences. Efforts to find effective treatments for recurrent meningiomas have remained elusive. Higher WHO-grade meningiomas have exhibited greater Programmed Death Ligand 1 (PD-L1) expression through various methods, but the prognostic value of PD-L1 expression has not been described in the context of molecular profiling. Additionally, trials investigating PD-1/PD-L1-targeted immunotherapies have produced disappointing results. Here, we find that PD-L1 positivity, while prevalent in MenG C tumors, does not predict recurrence in the benign MenG A and B tumors. PD-L1 positivity also occurs independently of CDKN2A/B loss, a core component of the WHO grade that is regularly used in clinical trial selection criteria. Our results indicate that future clinical trials for PD-1/PD-L1 centric immunotherapies should select patients after molecularly profiling tumors to confirm aggressive MenG C status.

Meningiomas are the most common primary intracranial neoplasms.^1^ In recent years, we and others have used molecular profiling to identify 3 molecular groups, one of which, MenG C, is aggressive.^2-5^ Half of patients with MenG C tumors will recur within 47 months of treatment.^5^^,^^6^ Currently, surgery and radiotherapy are the only 2 treatment options, both of which fail to adequately treat MenG C tumors.^5^^,^^7^

Inhibition of Programmed Cell Death 1 (PDCD1, PD-1), its ligand (CD274, PD-L1), and its signaling cascade is used to treat multiple cancer types and can substantially improve patient survival.^8^ Higher-grade meningiomas tend to have higher expression of PD-L1 through various methods,^9^ prompting clinical trials to evaluate PD-1/PD-L1 antagonists in meningioma. However, for unclear reasons, the 2 clinical trials evaluating PD-1 or PD-L1-targeted immunotherapies have shown disappointing results, with a few case reports postulating the influence of tumor mutational burden in PD-1/PD-L1 inhibitor efficacy.^10-13^ It is currently unknown how PD-L1 positivity fits into the context of molecular classification, how PD-L1-positive tumors are distributed in the WHO grading system, and whether PD-L1 positivity continues to predict worse outcomes when considering established biomarkers of aggressive meningiomas, most notably CDKN2A/B loss.^6^ Taken together, this suggests a necessity for re-evaluating the utility of PD-L1 positivity as a biomarker and therapeutic opportunity for meningioma patients.

Towards this end, we examined 260 tumors for PD-L1 expression through immunohistochemistry with the 22C3 antibody using immunoreactivity intensity, tumor proportion score and/or a combined proportion score of 1 or greater^14^ (Figure 1A, Figure S1, Table S2). Out of 260 tumors, 33 tumors (12.7%) were considered positive (Table S1). In addition, we used RNA sequencing to molecularly classify the tumors as MenG A (benign, merlin-intact), MenG B (benign, merlin-lost), or MenG C (aggressive) as previously described.^15^

**Figure 1.**
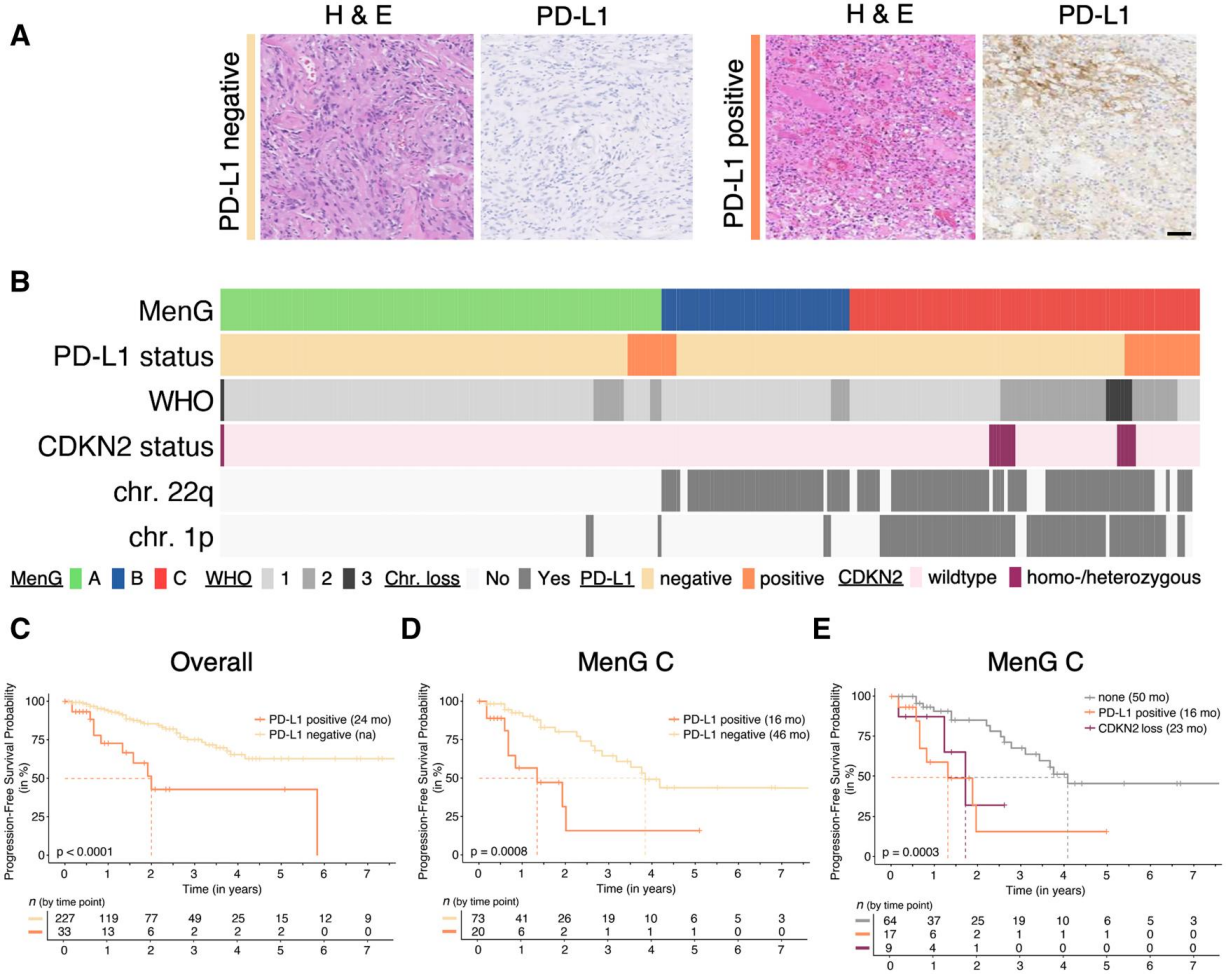
PD-L1 shortens progression-free survival (PFS). **A)** Examples of PD-L1 scoring based on immunohistochemistry staining for PD-L1 expression using the 22C3 monoclonal antibody. Scale bar = 100 µm. **B)** Oncoprint of 260 tumors with clinical data, clustered by molecular groups. **C–E)** Progression-free survival (PFS) of PD-L1 positive and negative tumors in this cohort **(C)**, in MenG C tumors only **(D)**, and in combination with CDKN2A/B status within MenG C **(E)**. Median PFS (dashed lines) are given in months. Pairwise log-rank test.

Of the 33 PD-L1 positive tumors, 9 (27.3%) were classified as MenG A, 4 (12.1%) as MenG B, and 20 (60.6%, *P *= .0084) as MenG C (Figure 1B). Overall, PD-L1 positive tumors had significantly worse progression-free survival (that is re-growth after subtotal resection or recurrence after gross total resection) compared with tumors that were negative (*P *< .0001, Figure 1C). There was no difference in extent of resection (*P *= .306) or treatment with adjuvant radiotherapy (*P *= 1) between PD-L1 positive and negative tumors (Table S1). Interestingly, when looking in the context of molecular groups, PD-L1 positivity in MenG C tumors resulted in a significantly shorter median progression-free survival (mPFS) of 16 months, compared to 46 months for all MenG C tumors (*P *= .0008, Figure 1D). This was not seen with MenG A or MenG B PD-L1 positive tumors (Figure S2A and SB). PD-L1 positivity in the context of WHO grade did not reveal this highly aggressive group (Figure S2C and SD).

Since we know that the subset of MenG C tumors with CDKN2A/B loss has an even worse prognosis, we wanted to understand whether PD-L1 positivity identifies the same subgroup.^6^ Thus, we analyzed our MenG C tumors to identify the 12 tumors with either heterozygous or homozygous CDKN2A/B loss in our cohort. The CDKN2A/B and PD-L1 groups are largely exclusive (*P *= .7166, Fisher’s exact test) as 9 of 12 CDKN2A/B-loss MenG C tumors (76.9%) were PD-L1 negative and 17 of 20 PD-L1 positive MenG C tumors (85.0%) were CDKN2A/B intact. This is also illustrated in the recurrence-free survival of each group within MenG C tumors (Figure 1E). The 3 MenG C tumors that exhibited concurrent CDKN2A/B loss and PD-L1 positivity had even worse outcomes. All 3 patients had multiply-recurrent tumors, 1 being diagnosed at 24 years old, 1 passing away from excess tumor burden 6 months post-surgery, and another recurring within 2 months after resection.

Taken together, these results suggest PD-L1 positivity is an independent prognostic factor for meningioma aggressiveness in the framework of MenG classification. It adds crucial, non-overlapping clinical information, even in the context of CDKN2A/B loss and molecular classification. Routine PD-L1 staining following surgery can be performed in any standard laboratory with immunostaining capabilities and would be beneficial for post-operative treatment planning when combined with molecular classification.

An interesting discordance between our study and previous studies investigating PD-L1 as a prognostic biomarker is that we have found a far lower proportion of high WHO grade meningiomas to express PD-L1.^16^^,^^17^ A possible reason for this discrepancy is our use of a PD-L1 22C3 antibody as opposed to prior use of SP142. Studies have shown that SP142 and 22C3 have low concordance and that SP142 is a less reliable marker for PD-L1 compared to 22C3 in other cancers.^18^ The efficacy of these markers in detecting PD-L1-positive meningiomas should be evaluated in future studies.

An important limitation of our study is the incorporation of primary and recurrent tumors within our cohort. Recurrent status itself is known to affect PD-L1 expression and future recurrence.^19^ Future studies should aim to analyze larger cohorts and conduct subgroup analyses to determine the independent prognostic effect of PD-L1 positivity in primary MenG C tumors.

In summary, our study analyzed a cohort of consecutive meningioma cases (treated by the corresponding author), revealing that over 20% of MenG C tumors exhibit PD-L1 positivity. This finding highlights a potentially significant subset of hyperaggressive meningiomas that may benefit from PD-1/PD-L1 targeted therapies. One possible explanation for the mixed results of previous clinical trials^10^^,^^11^ could be that they did not molecularly classify the tumors, even though many were likely MenG C based on their aggressive nature. Additionally, using WHO grade as a screening criterion is inadequate, as 75% of high-grade tumors in our cohort are PD-L1 negative, limiting the likelihood that patients will respond to therapy. By refining patient selection to include both molecular classification and PD-L1 expression, future trials may demonstrate stronger evidence for the efficacy of PD-1/PD-L1 blockade in meningioma treatment.

## Supplementary Material

pkag023_Supplementary_Data

## Data Availability

De-identified individual participant pathology can be shared upon reasonable request to the corresponding author (T.J.K. and A.J.P.). RNA-sequencing data are available on the Gene Expression Omnibus (GSE136661, GSE189672).
