# Supplementary material for: PD-L1 positivity predicts a unique hyperaggressive tumor group within MenG C meningiomas
Source: JNCI Cancer Spectr. 2026 Mar 7;10(2):pkag023. doi: 10.1093/jncics/pkag023 (PMC13032903; doi:10.1093/jncics/pkag023)
Supplement: pkag023_Supplementary_Data [file pkag023_supplementary_data.docx]

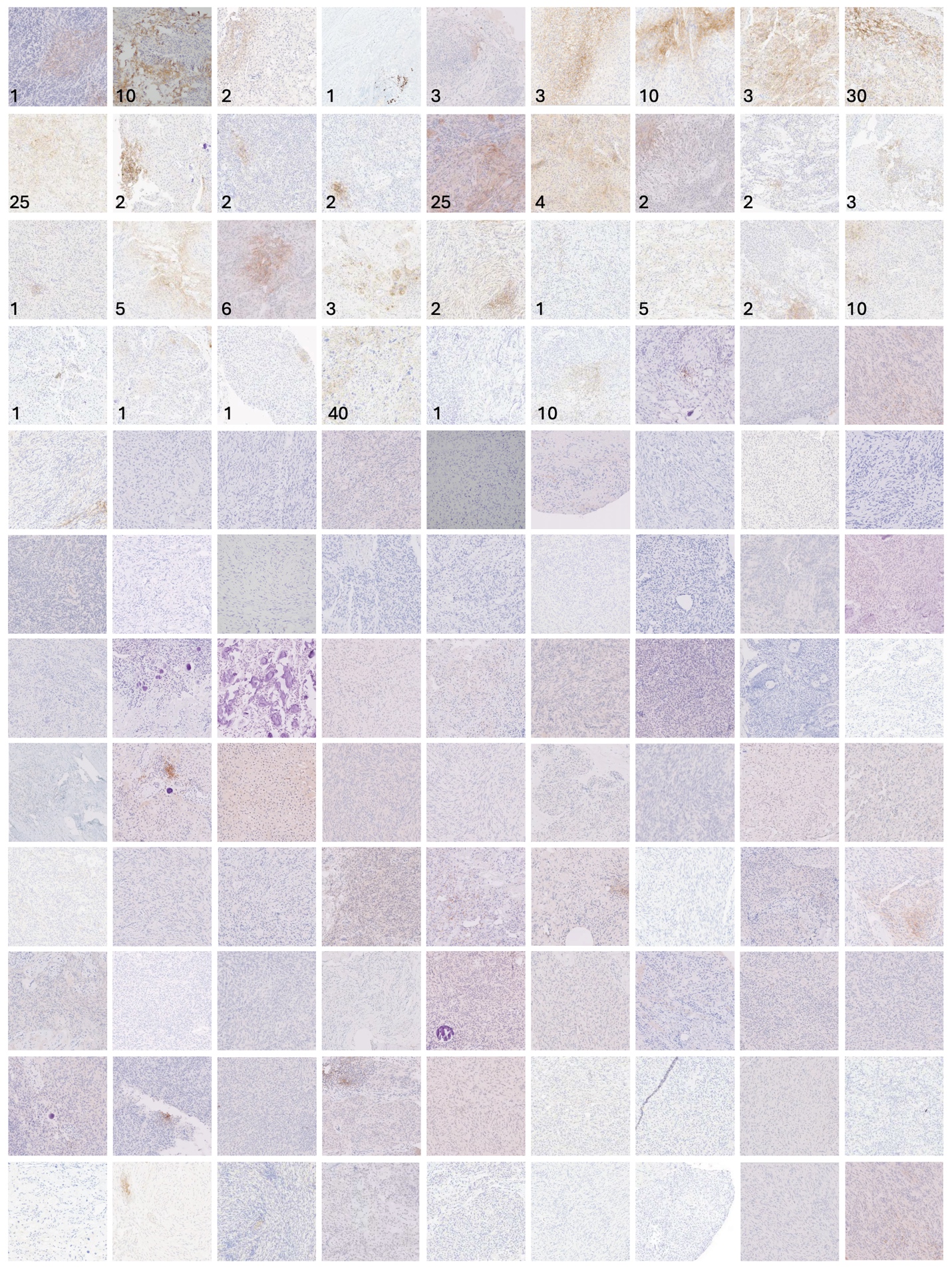

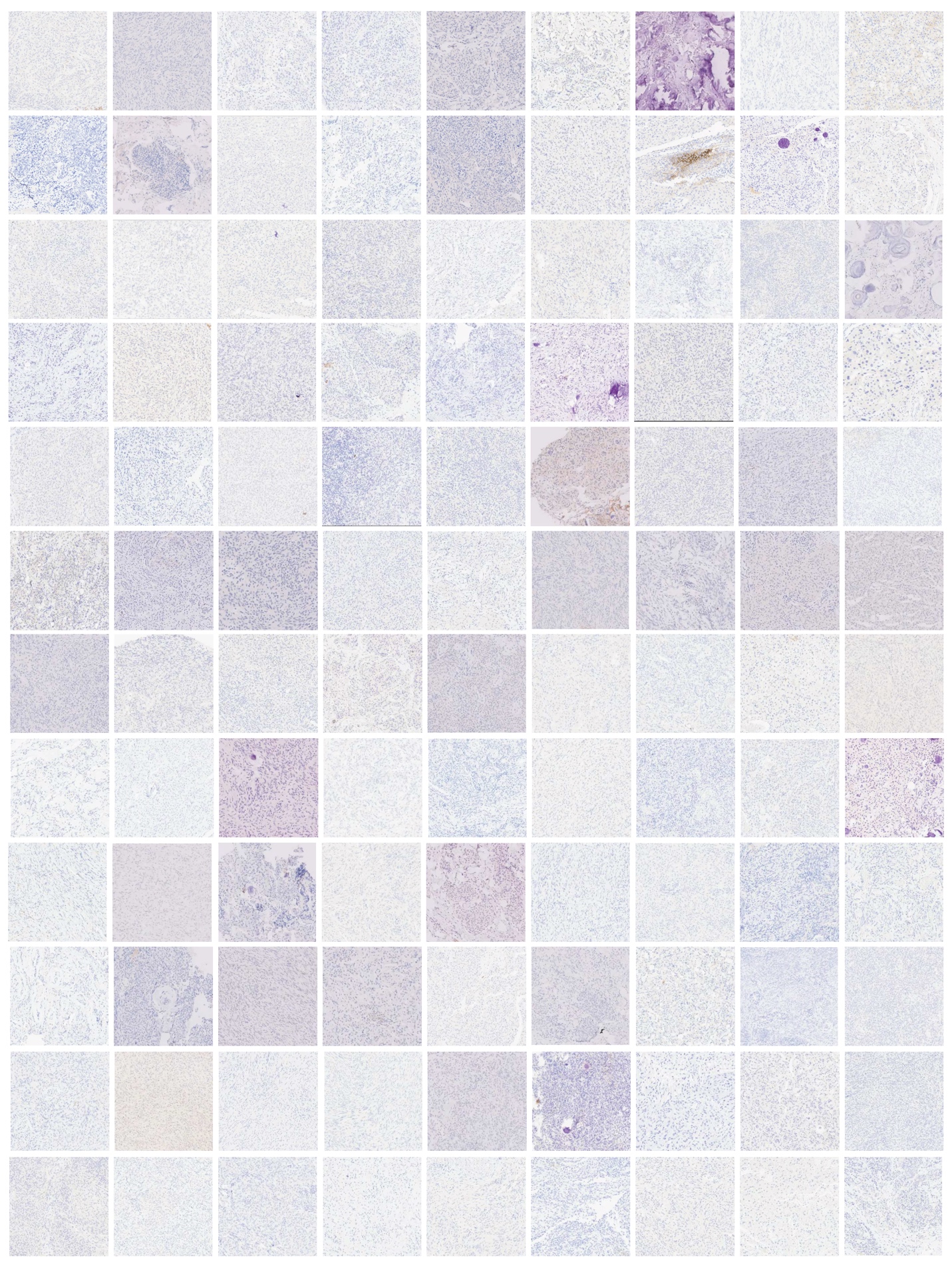

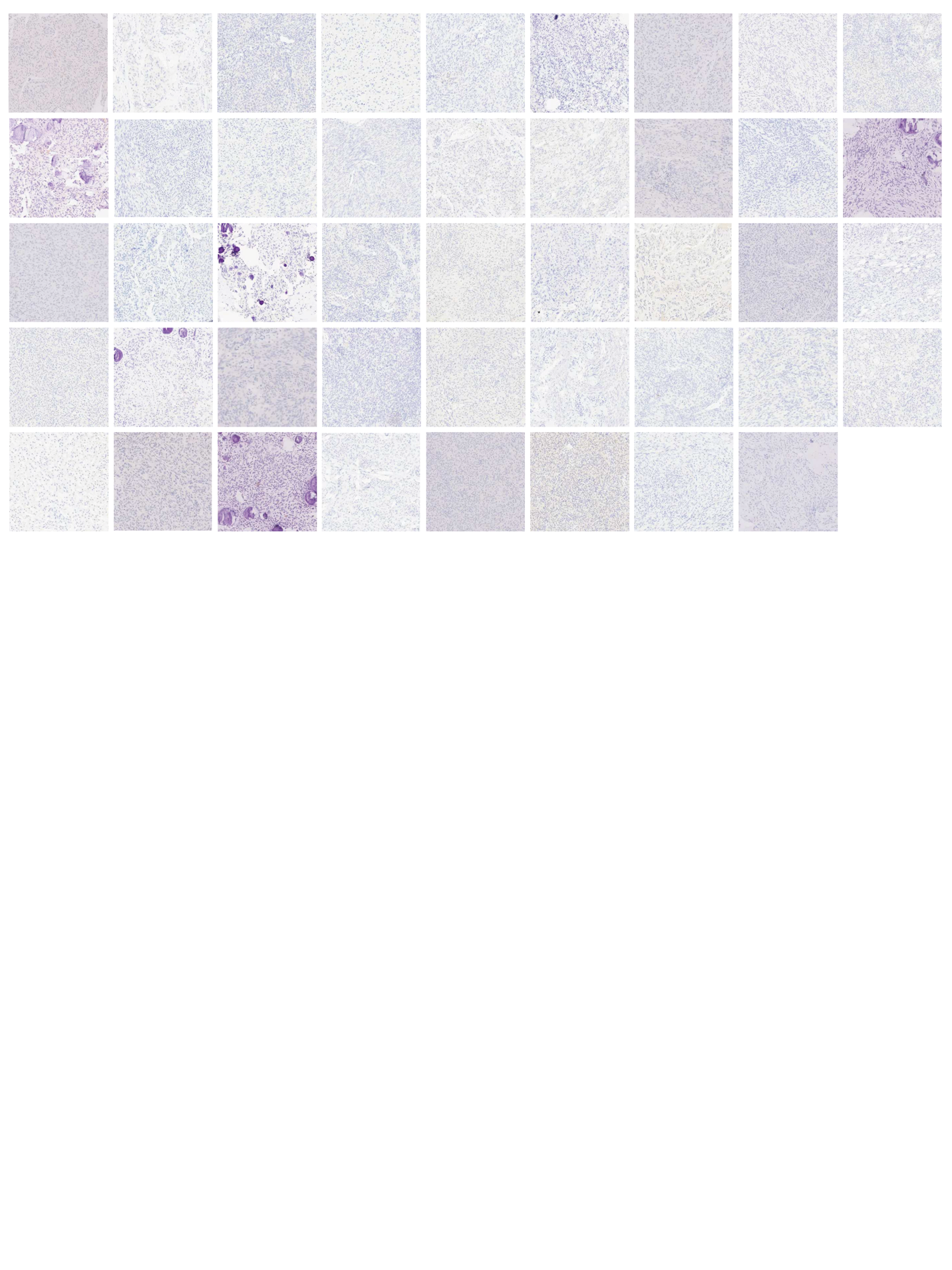
Figure S1. Immunohistochemistry staining of all tumors using 22C3 monoclonal antibody. Combined proportion score of 1 or greater is shown.


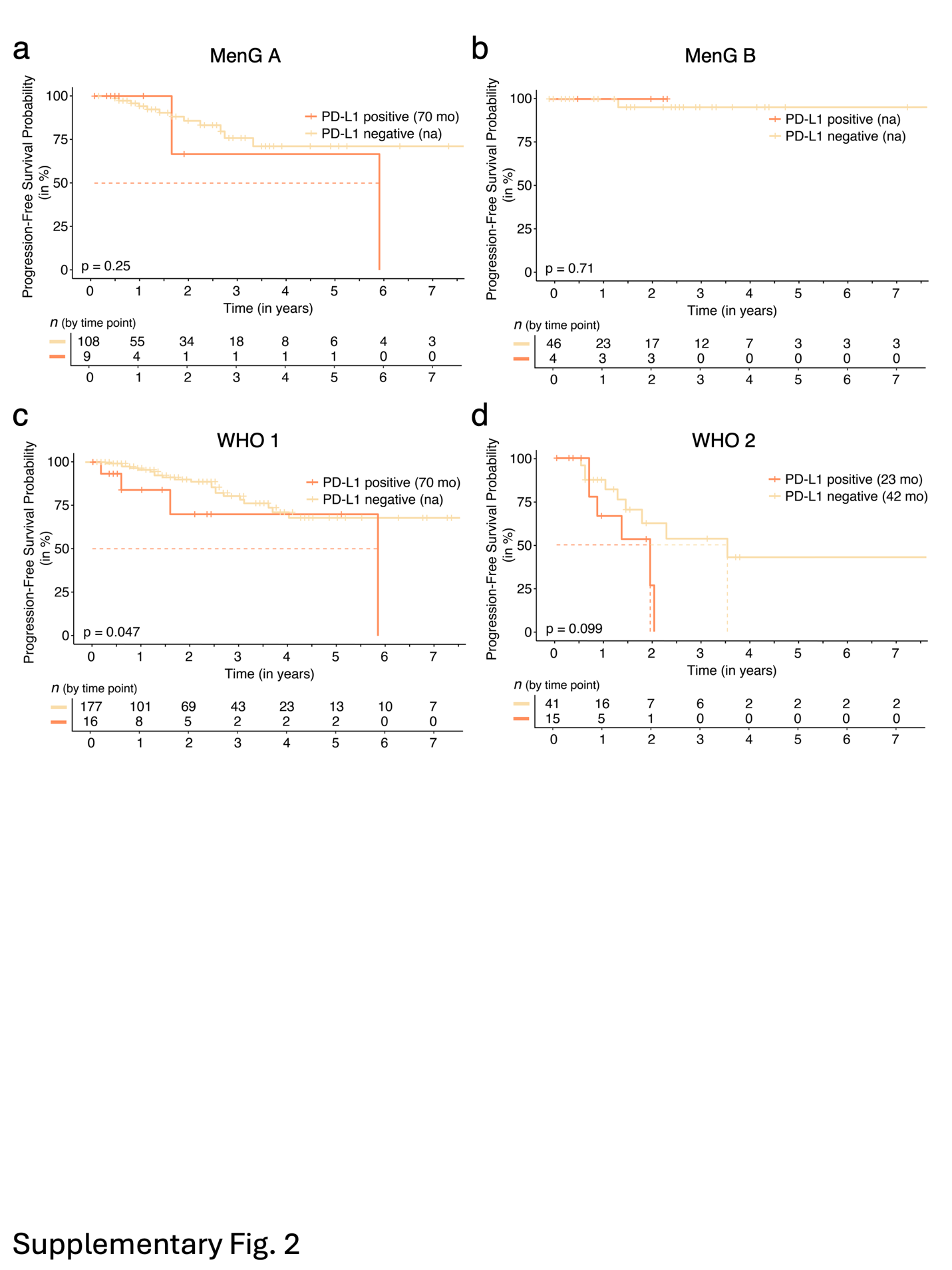
Figure S2. Progression-free survival (PFS) of PD-L1 positive and negative tumors in MenG A (a), MenG B (b), WHO 1 (c), and WHO 2 grade tumors (d). median PFS (dashed lines) are given in months. Pairwise log-rank test.

|  | PD-L1 Negative (n = 227) | PD-L1 Positive (n = 33) | p-value |
| --- | --- | --- | --- |
| Molecular Group |  |  | 0.006 |
| A | 108 (47.6) | 9 (27.3) |  |
| B | 46 (20.3) | 4 (12.1) |  |
| C | 73 (32.2) | 20 (60.6) |  |
| Chromosome 22q loss | 103 (45.8) | 17 (51.5) | 0.667 |
| Chromosome 1p Loss | 64 (28.3) | 14 (42.4) | 0.148 |
| Sex |  |  | 0.266 |
| Female | 163 (71.8) | 20 (60.6) |  |
| Male | 64 (28.2) | 13 (39.4) |  |
| Age | 58.65 (13.60) | 58.03 (17.50) | 0.874 |
| Tumor |  |  |  |
| Primary | 197 (86.8) | 23 (69.7) | 0.022 |
| Recurrent | 30 (13.2) | 10 (30.3) |  |
| Pathology |  |  | 0.002 |
| Indeterminate | 2 (0.9) | 0 (0.0) |  |
| WHO I | 177 (78.0) | 16 (48.5) |  |
| WHO II | 41 (18.1) | 15 (45.5) |  |
| WHO III | 7 (3.1) | 2 (6.1) |  |
| Brain Invasion | 15 (6.6) | 7 (21.2) | 0.013 |
| Necrosis | 34 (15.0) | 7 (21.2) | 0.515 |
| Extent of Resection |  |  | 0.306 |
| Partial | 85 (37.4) | 16 (48.5) |  |
| Total | 142 (62.6) | 17 (51.5) |  |
| Adjuvant Radiotherapy | 23 (10.1) | 3 (9.1) | 1 |
| Recurrence | 33 (14.5) | 11 (33.3) | 0.015 |
| CDKN2A/B Loss | 10 (4.4) | 3 (9.1) | 0.472 |

Table S1*.* Summary Characteristics. Note: Categorical variables were described using n (%), while continuous variables were described using mean (standard deviation). Chromosome 22q and 1p loss were determined by a 33% threshold. CDKN2A/B loss includes both homozygous and heterozygous deletions. P-values were determined by Wilcoxon rank sum tests for continuous variables and Chi-square/Fisher’s exact tests for categorical variables.

| Tumor Proportion Score | Combined Proportion Score |
| --- | --- |
| 0 | 1 |
| 0 | 1 |
| 0 | 1 |
| 0 | 1 |
| 1 | 1 |
| 1 | 1 |
| 1 | 1 |
| 0 | 2 |
| 0 | 2 |
| 0 | 2 |
| 1 | 2 |
| 2 | 2 |
| 2 | 2 |
| 2 | 2 |
| 2 | 2 |
| 3 | 3 |
| 3 | 3 |
| 3 | 3 |
| 3 | 3 |
| 0 | 4 |
| 5 | 5 |
| 5 | 5 |
| 5 | 5 |
| 4 | 6 |
| 10 | 10 |
| 10 | 10 |
| 10 | 10 |
| 3 | 10 |
| 25 | 25 |
| 25 | 25 |
| 30 | 30 |
| 40 | 40 |

Table S2. Tumor Proportion and Combined Proportion Score of all Positive PD-L1 Samples (n = 33)
